# Investigation of the emergency water supply schemes for youkou groundwater source field in Nanchang using a simulation–optimization model

**DOI:** 10.1038/s41598-024-52303-0

**Published:** 2024-01-31

**Authors:** Qingshan Ma, Huijie Wu, Weiya Ge, Jia Zhang

**Affiliations:** 1 Nanjing Center, China Geological Survey, Nanjing, 210016 Jiangsu China; 2 Hydrogeology Team of Hebei Coalfield Geology Bureau, Handan, 056000 Hebei China; 3 School of Water Resources and Environment, China University of Geosciences (Beijing), Beijing, 100083 China

**Keywords:** Environmental impact, Environmental sciences

## Abstract

To enhance the resilience of Nanchang’s water supply system and ensure a dependable emergency water supply. Taking youkou emergency groundwater source field as an example, a flow simulation model was developed through an analysis of the hydrogeological conditions in the study area. Additionally, an optimization model based on the genetic algorithm (GA) technique was constructed and integrated into the flow simulation model. Subsequently, various water supply schemes were simulated with the minimum cost of groundwater extraction as the objective function. The results show that the values of the objective function were reduced by 4.92%, 15.67%, and 42.35% for the three different optimization schemes, namely pumping rates, joint pumping rates and the number of wells, and joint pumping rates, number of wells and well location. Ultimately, the optimal emergency water supply scheme was determined by considering a comprehensive range of factors. These factors encompassed considerations such as the area of the water level depression funnel, the dewatering thickness of the aquifer and the recovery of the groundwater level. The practice shows that the simulation–optimization model could effectively simulate complex groundwater flow systems, meeting the objective function and constraints toachieve the optimal exploitation scheme.

## Introduction

With rapid urbanization and industrialization, water scarcity has emerged as a critical factor inhibiting urban development and threatening people’s livelihoods^1^. Notably, the significance of urban water supply is progressively escalating, assuming a pivotal role in meeting the essential human requisites. Due to the distinctive continental monsoon climate prevalent in China, the distribution of water resources exhibits a highly uneven pattern. It means that cities that relying on surface water sources are at risk of a water crisis during dry seasons. Furthermore, the quality of surface water is highly susceptible to pollution, which further exacerbates variability in water supply^2^.

Nanchang, the capital of Jiangxi Province, is characterized by its substantial potential for economic development. And the surface water serves as the primary source of water supply. However, the single water resource model is insufficient to meet increasing water consumption, and posing a significant threat to the smooth operation of the city. Historical data reveal that Nanchang has experienced multiple severe droughts over the last few years^3,4^. In comparison to surface water, groundwater has superior water quality, stable dynamics, and a better spatial and temporal distribution, making it an ideal emergency water source. Therefore, studying the emergency capacity of groundwater is critical for improving cities’ ability to manage emergencies and ensure the safety of their water supplies.

In recent years, the awareness of urban water crisis prevention has grown both domestically and internationally due to various natural disasters and man-made emergencies. As a result, many experts and scholars have been concerned with emergency water supply and emergency water source planning^5–7^. Presently, the integration of optimization algorithms with groundwater flow models to address issues in groundwater resource management has emerged as a prominent subject of research^8–11^. For instance, Akbarpour et al.^12^ employed various algorithms, including genetic, particle swarm, and firefly algorithms, to optimize the pumping strategy of a hypothetical aquifer. Similarly, Ghaseminejad and Shourian^13^ coupled a particle swarm algorithm with MODFLOW to determine the optimal location for a pumping well and flow rate that minimizes the costs related to drilling, delivery, and water treatment, ultimately reducing the cost of water extraction. Other researchers have used a combination of different optimization methods to address optimal groundwater resource management, water resource allocation, and groundwater management models^14–16^. However, there is still a limited application of coupling intelligent optimization algorithms with groundwater flow models for designing the location of groundwater pumping facilities and pumping schemes in China.

The objective of this study is to formulate a scientifically sound emergency water supply scheme. This work involves the construction and calibration of a transient simulation model that accurately represents groundwater flow dynamics. The established flow model is subsequently integrated with an optimization model founded on genetic algorithms. Through this synergy, the research seeks to identify optimal pumping strategies that align with existing water demands while simultaneously minimizing the overall withdrawal expenses. These expenses encompass construction, operational, and environmental costs. The outcomes of this research endeavor hold the potential to provide a robust scientific foundation for the development of groundwater-based emergency water sources in Nanchang.

## Overview of the study area

**Figure 1:**
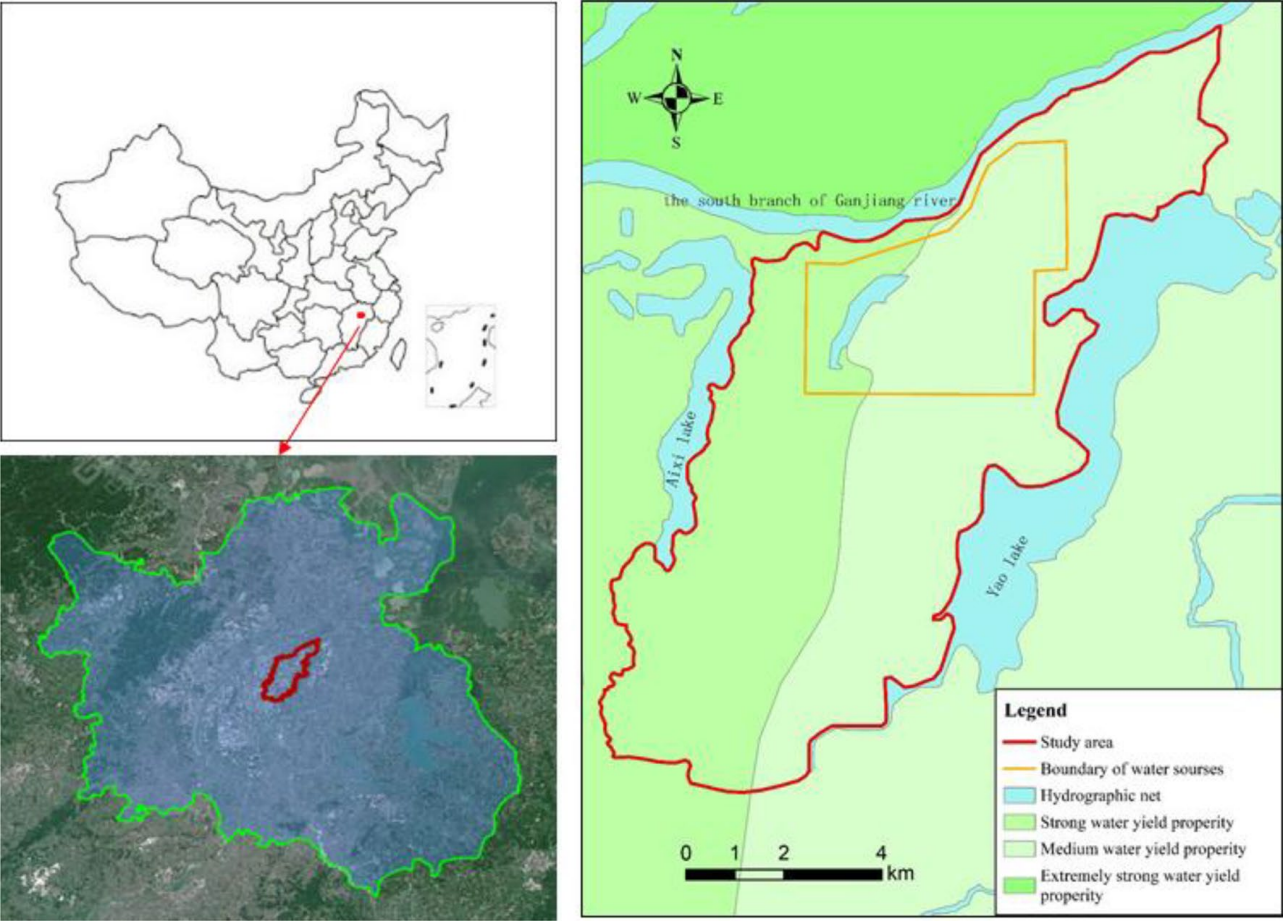
General view of the study area.

The study area lies within the Ganfu plain, spanning 28.62–28.77° N latitude and 115.96–116.09° E longitude. And the youkou groundwater source area is situated in the northern part of the study area, in proximity to the South Branch of the Ganjiang River (Fig. 1). The area is about 84.776 km^2^, and the terrain is flat and characterized by a humid subtropical climate with extended, moist summers and brief, cool winters. The annual average temperature ranges between 17 and 17.7 °C, with historical extremes from − 9.7 °C on December 29, 1991 to 40.6 °C on July 23, 1961. Annual precipitation averages about 1610 mm, with 49.33% concentrated in April to June. Average annual evaporation reaches 1227.4 mm.

The area exhibits a significant presence of Quaternary deposits, with a thickness ranging from 20 to 30 m, and the groundwater is mainly composed of loose pore water. The majority of the aquifers within the study area have a binary structure, the sand and gravel layer underlying the surface layer functions as the primary aquifer. This aquifer has a thickness of 10–20 m and abundant water resources, with a hydraulic conductivity value of about 53–161 m/day. The groundwater level generally ranges from  9 to 15 m, with water level changes spanning between 3 and 5 m. The pore water level changes are highly dependent on atmospheric precipitation, with levels rising during the rainy season and decreasing during the dry season.

**Figure 2:**
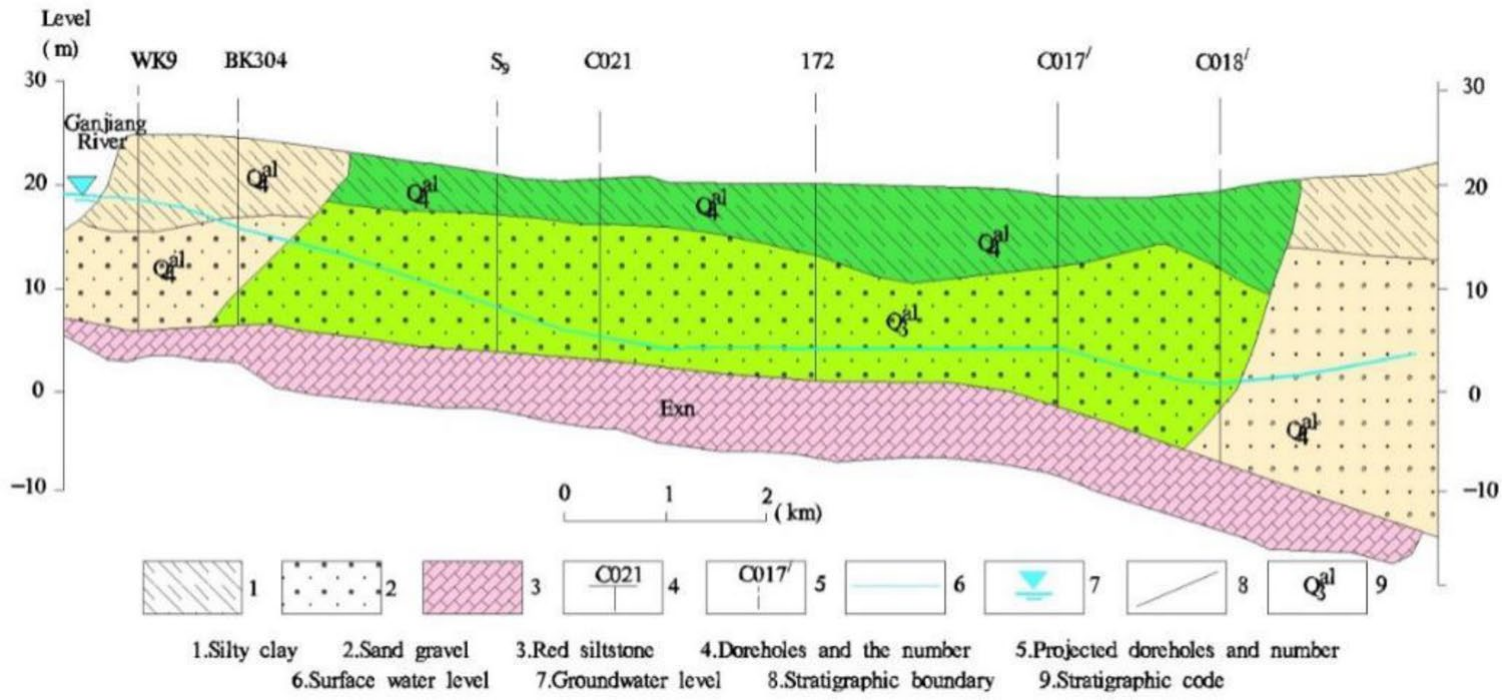
Hydrogeological profile of the study area.

The red bed aquifers are widely concealed below the Quaternary loose soil layer, with elevations ranging from − 30 to − 60 m. These aquifers possess excellent connectivity and form a continuous and uniform confined water level. Due to long-term weathering damage, fissures are relatively developed and have certain water permeability, resulting in cross-flow connections between the “red bed” aquifer and the overlying loose pore aquifer^17^. A visual representation of the study area’s hydrogeological profile is displayed in Fig. 2.

## Methodology

The simulation–optimization approach unfolded in two distinct phases. Initially, a meticulous construction and calibration of a transient groundwater flow model were undertaken to assess the present water budget and simulate hydrological responses to diverse groundwater pumping schemes. The subsequent phase involved developing an optimization model utilizing the GA technique. This model was then integrated with the simulation model to search for global optimal or near-optimal solutions while adhering to management objectives and relevant constraints^18^.

An iterative procedure was established, where the optimization model invokes the simulation model (MODFLOW) to validate constraints and evaluate the objective function in each iteration. This iterative process continues until a predefined convergence criterion is met^18^.

### Flow numerical model

The MODFLOW-2000 code was used to simulate the groundwater flow^18,19^. MODFLOW is a block-centered finite difference code that imitates saturated flow in two or three dimensions with various boundary conditions. The model was calibrated utilizing the automated inverse modeling code PEST^20^.

#### Boundary conditions

The numerical model covers an area of approximately 84.78 km^2^. The northern boundary of the model is the southern branch of the Ganjiang River, which has a relatively stable water level. Furthermore, the hydrogeological survey results in the study area indicate that the riverbed of the southern branch of the Ganjiang River contains Upper Pleistocene or Holocene silty clay and muddy clay, and there is a hydraulic connection between the river and groundwater. Therefore, the river boundary conditions were applied along the northern boundary, connecting the aquifer to the south branch of the Ganjiang river. Additionally, general-head boundary conditions were assigned along the eastern and western lakes, as well as the southern administrative boundary. Recharge was introduced at the model’s top to account for both natural recharge (computed as precipitation minus runoff and evapotranspiration) and estimated agricultural irrigation water leakage. The model’s base was defined as a no-flow boundary.

#### Spatial and temporal discretization

**Figure 3:**
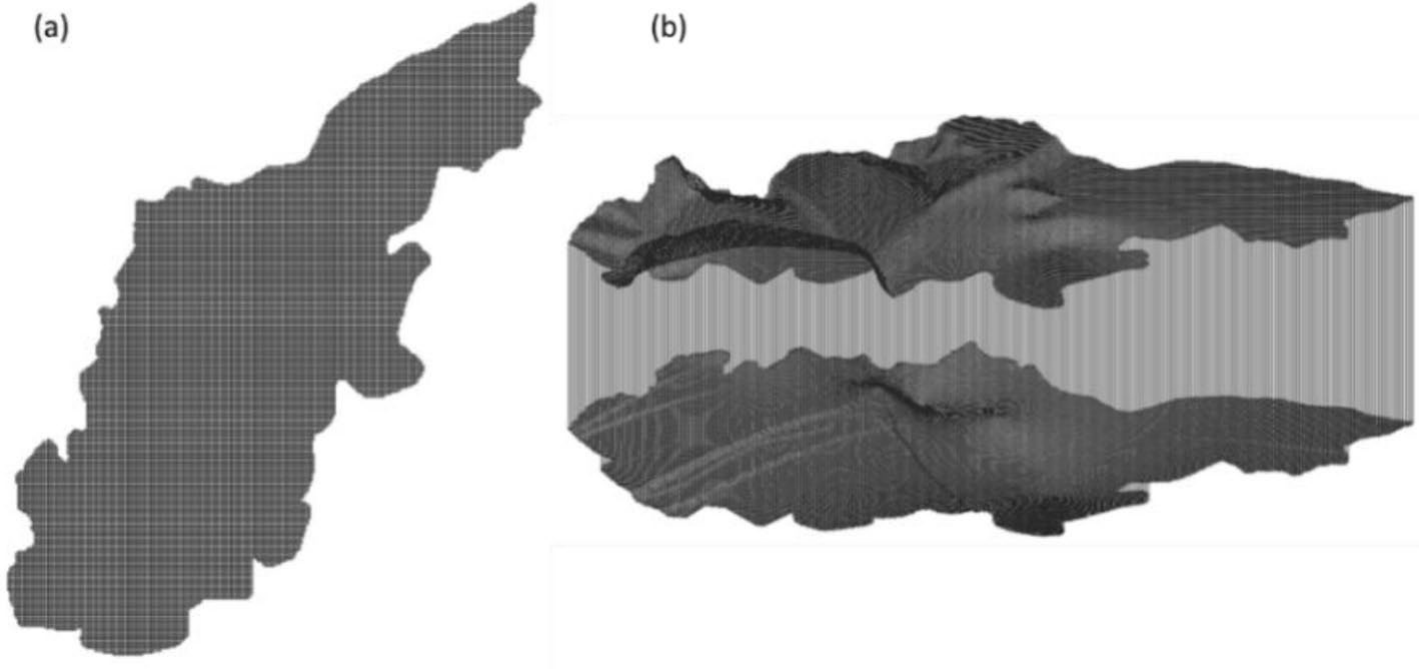
Schematic diagram of grid division.

The simulated region was discretized by a horizontal grid of 315 rows and 258 columns, featuring a uniform spacing of 50 m. Vertically, the model comprises one layer with an average thickness that ranges between 22 and 40 m (Fig. 3). The upper and lower elevations of this layer for each individual model cell were determined through the application of a digitized topographic map and thickness contour maps^18^.

The simulation model utilized in this study aimed to assess transient pumping from 2017 to 2019. Considering the available data and the groundwater level dynamics, each month was treated as an individual stress period within MODFLOW, further subdivided into three time steps. Initial head values for January 2017 were derived from water level data.

#### Hydrogeological parameters assignment

**Figure 4:**
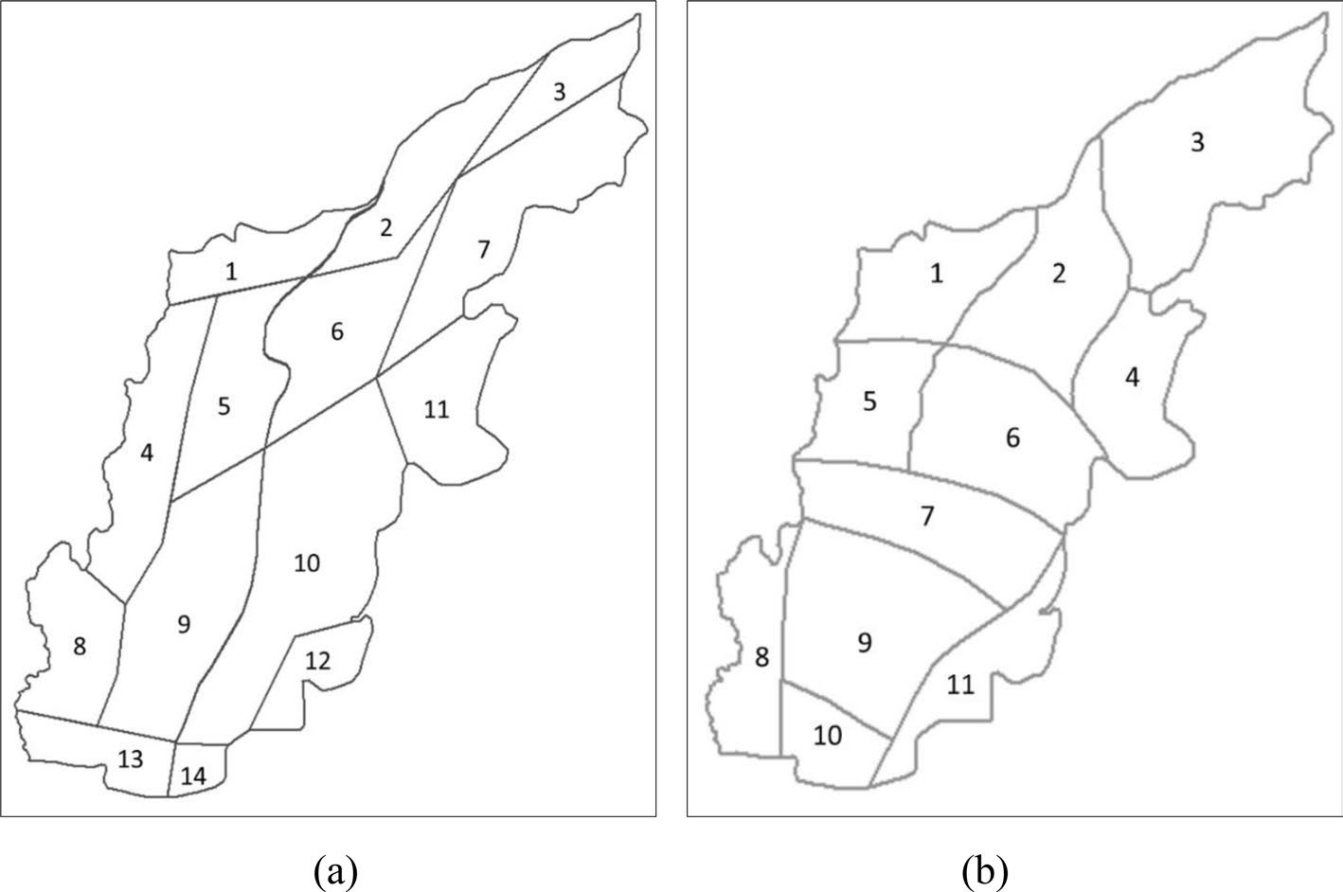
Hydrogeological parameter zoning map: (**a**) hydraulic conductivity; (**b**) precipitation infiltration coefficient.

The results of hydrogeological survey show that the Quaternary pore aquifer in the study area is mainly composed of sand and gravel layers from the Holocene, Upper Pleistocene, and Middle Pleistocene. And the hydraulic conductivity generally ranges from 53 to 161 m/day. Based on geological heterogeneity and results of aquifer tests, the model domain was divided into 14 zones for the parameterization of hydraulic conductivity ($K$) and 11 zones for the parameterization of precipitation infiltration coefficient ($\alpha$), as shown in Fig. 4 and Table 1. The zonal $K$ values ranging from 50 to 150 m/day and $\alpha$ values ranging from 0.15 to 0.4.

**Table 1:** The values of hydrogeological parameters assignment.

Zone	$K$ (m/day)	$\alpha$	Zone	$K$ (m/day)	$\alpha$
1	90	0.25	8	150	0.3
2	120	0.15	9	140	0.4
3	90	0.2	10	120	0.35
4	80	0.15	11	60	0.3
5	100	0.15	12	130	–
6	110	0.2	13	100	–
7	70	0.25	14	90	–

#### Model calibration

**Figure 5:**
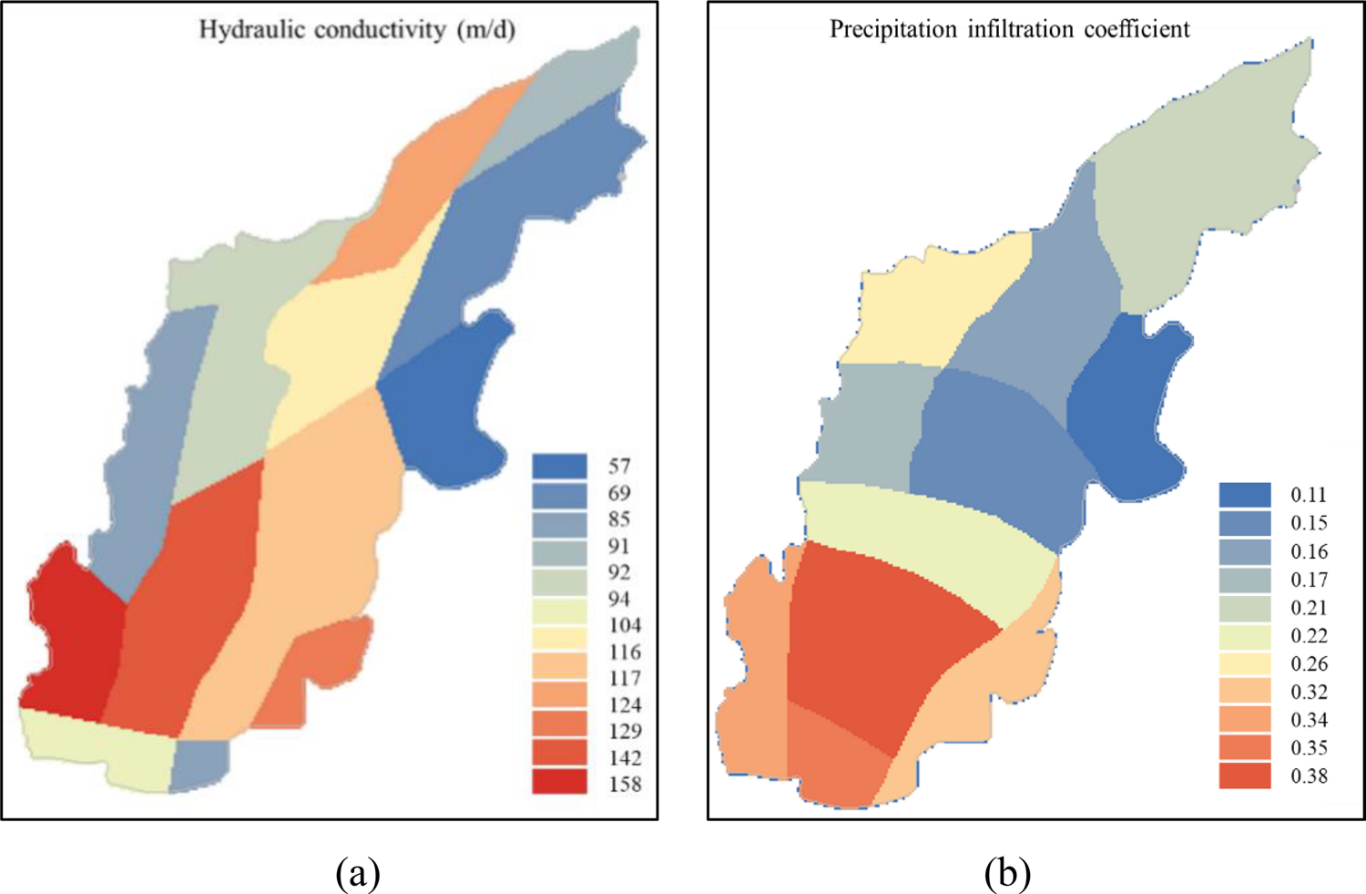
The model parameter partition: (**a**) hydraulic conductivity; (**b**) precipitation infiltration coefficient.

In this investigation, the automated parameter estimation code PEST was implemented to adjust hydraulic parameter values until achieving an optimal match between observed and simulated water levels. Parameters undergoing automatic calibration encompassed hydraulic conductivity, general head boundary (GHB), recharge, and specific storage^18^. The calibrated hydraulic conductivity of the aquifer is visualized in Fig. 5.

**Figure 6:**
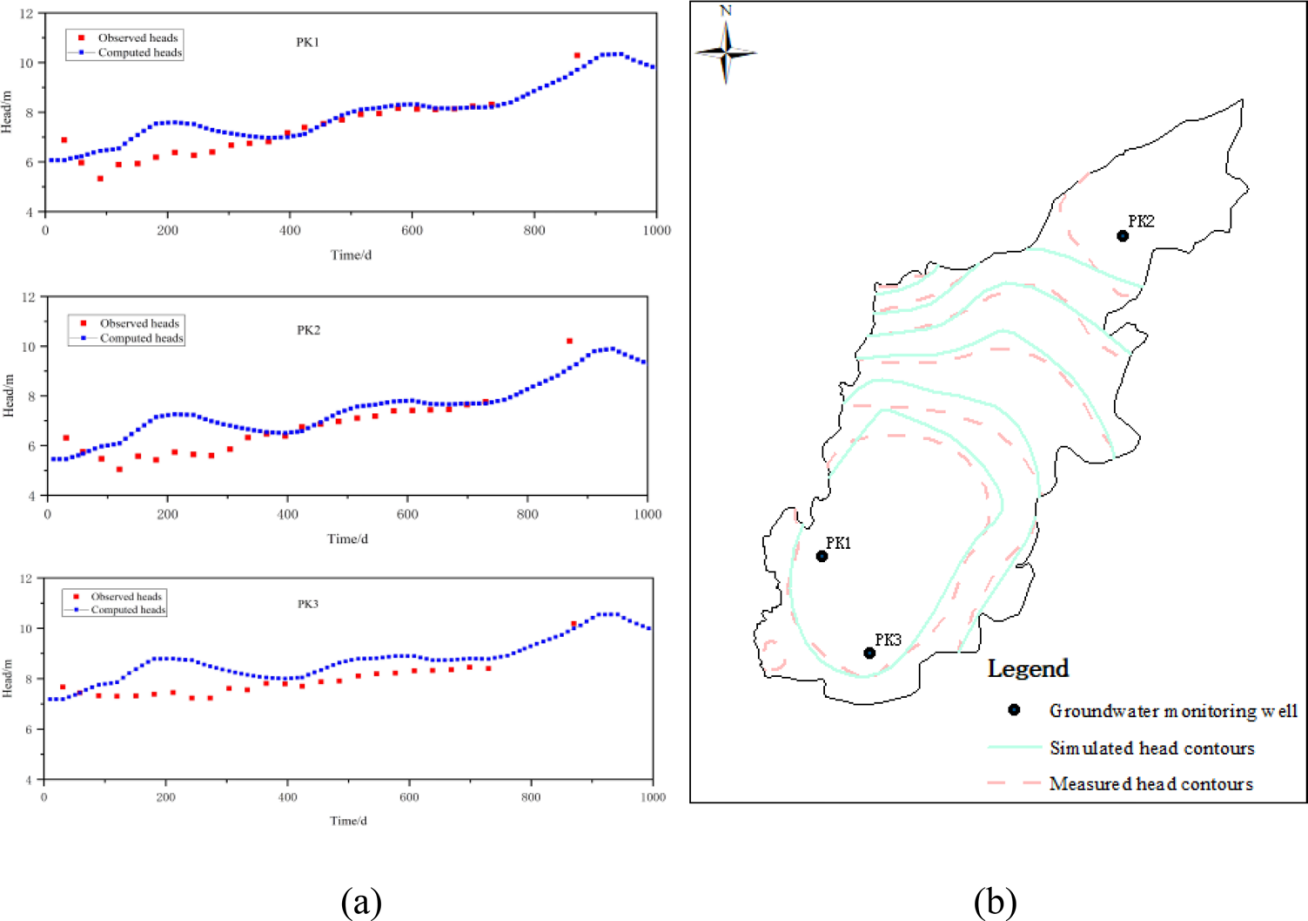
(**a**) Groundwater level for typical observation wells in the calibration period; (**b**) fitting curves of the groundwater flow field in the verification period.

To assess the calibrated model’s performance, simulated hydraulic heads were compared to measured water levels at PK1/PK2 and PK3 observation wells for the 2017–2018 periods. The calibration results are presented in Fig. 6a. It can be seen that there is good agreement between the observed and computed values. For the verification processes, groundwater flow field data from May 2019 were utilized to verify the model. A comparison between simulated and observed head contours is provided in Fig. 6b.

The resulting water budget at the end of the calibration period (December 2019) is presented in Table 2. The flow budget indicated that the major input term was the recharge, accounting for 45.5% of total inflow to the aquifer. On the output side, groundwater exploitation constitutes 61.55% of total outflow from the aquifer, making it the most significant component of the aquifer’s output.

**Table 2:** Computed water budget at the end of the calibration period (December 2019).

Water balance term	(× 10^3^m^3^/day)
Inflow	
Storage	6.657
Recharge	50.161
River leakage	3.636
Head DEP bounds	49.802
Total inflow	110.256
Outflow	
Storage	6.270
Wells	67.862
River leakage	9.784
Head DEP bounds	26.340
Total outflow	110.256

### Multi-objective optimization formulation

The present study aims to minimize costs via the objective function, which encompasses various cost factors such as total annualized drilling costs, transmission expenses, and environmental protection expenditure. To achieve this goal, the optimization of pumping rates, well locations, and the number of wells is quintessential. In this regard, the total annualized drilling cost includes drilling, casting, and equipment expenses, along with other relevant costs^21,22^. These can be expressed as the following:

1 $$f_{1} = U_{drilling} /T \times N_{active} ,$$

where $f_{1}$ is the total annualized drilling cost function; $U_{drilling}$ is the drilling cost of a single well; *T* is the operating time of the pumping well; $N_{active}$ is the number of pumping wells.

The operation of a pump to extract groundwater necessitates energy consumption to elevate the water to the surface. As a secondary goal, this study aims to minimize the energy expenditure involved in pumping from wells. Several key factors influence pumping cost, including the quantity of water to be lifted, its density, hydraulic head, pump efficiency, and the energy cost associated with pumping a given volume of groundwater per kilowatt-hour^13,21^. This study, however, excludes other costs from consideration. Consequently, the overall pumping cost can be expressed as:

2 $$f_{2} = \sum\limits_{i = 1}^{{N_{active} }} {\gamma_{w} } \cdot q_{i} \cdot h_{pi} /\eta \times \Delta t \times U_{\cos t} ,$$

where $f_{2}$ is the energy cost function; $N_{active}$ is the number of pumping wells; $\gamma_{w}$ is the specific weight of the groundwater; taken as 980 N·m^3^; $q_{i}$ denotes the single well pumping capacity of the $i$th well, taken as m^3^/d; $h_{p}$ denotes the head in m; $\eta$ denotes the pump efficiency, taken as 0.7; $\Delta t$ denotes the pumping time (length of each stress period)^21^; $U_{\cos t}$ denotes the cost of pumping per unit volume of groundwater per kilowatt hour, taken as 0.6/(3.6 × 10^5^) J.

The excessive pumping of groundwater may result in drawdown and worsen the issues of land subsidence, water quality degradation, and saltwater intrusion in the region, according to Yin and Tsai^23^. In order to ensure the lasting preservation of natural water resources and groundwater sustainability, it is imperative to maintain the groundwater level at or near an acceptable target level, thus reducing the impacts of groundwater pumping. The third objective is to minimize the variation in groundwater levels at the designated monitoring locations^12^.

3 $$f_{3} = \sum\limits_{i = 1}^{{N_{obs} }} {S_{i} } ,$$

where $f_{3}$ is the total depth of groundwater level drop at the observation wells in m; $N_{obs}$ is the number of observation wells; $S_{i}$ is the drawdown of groundwater at the location of the well $i$ in m.

The mathematical formula of the objective function is as follows^13^:

4 $$\left\{ \begin{array}{*{20}l} {\text{Minimize Z = }}\alpha_{{1}} f_{1} + \alpha_{{2}} f_{2} + \alpha_{{3}} f_{3} \hfill \\ f_{1} = U_{drilling} /T \times N_{active} \hfill \\ f_{2} = \sum\nolimits_{i = 1}^{{N_{active} }} {\gamma_{w} } \cdot q_{i} \cdot h_{pi} /\eta \times \Delta t \times U_{\cos t} \hfill \\ f_{3} = \sum\nolimits_{i = 1}^{{N_{obs} }} {S_{i} } \hfill \\ \end{array} \right..$$

Subject to:

$$I_{\min } \le I_{w} \le I_{\max } ,$$

$$J_{\min } \le J_{w} \le J_{\max } ,$$

$$q_{\min } \le q_{i} \le q_{\max } ,$$

$$N_{min} \le N_{active} \le N_{max} ,$$

$$Q = \sum\limits_{i = 1}^{{N_{active} }} {q_{i} } \ge Q_{min} ,$$

$$S_{i} < h_{0} .$$

In the above equations, $\left( {x_{i} ,y_{i} } \right)$ is the coordinates of the well $i$ in the MODFLOW model^13^, $\left( {I_{min} ,I_{max} } \right)$ is the longitudinal domain of search space, $\left( {J_{min} ,J_{max} } \right)$ is the latitudinal domain of search space, $q_{\min }$ and $q_{\max }$ are, respectively, the minimum and maximum allowable pumping rates in m^3^/day, $N_{min}$ and $N_{max}$ are the minimum and maximum number of wells, $q_{i}$ is the pumping rates of the $i$th well in m^3^/day, $i$ is the counter variable^13^. α_1_, α_2_ and α_3_ are the weight coefficients used to eliminate the influence of different dimensions of the objective function. If the total water supply is evenly distributed to 30–60 wells, the sum of the total annualized drilling costs and transmission expenses is about 110,000–130,000 yuan, and the environmental cost is about 350–450 m. According to the mean method^24^, α_1_ = 1/300, α_2_ = 1/300, and α_3_ = 1.

The management model described above presents a nonlinear challenge, which can be effectively addressed through various optimization approaches. Traditional methods like nonlinear programming or gradient techniques might not be the best options, as they would get stuck in local optimal solutions due to the inherent non-convexity of the unconfined groundwater problem. To circumvent these challenges, a Genetic Algorithm (GA) scheme is suggested^18^.

### Linkage between simulation model and GA-based optimization model

The genetic algorithm, developed by Holland^25^, is an optimization technique employing natural selection mechanisms to seek optimal solutions for intricate problems^26^. GA possesses notable advantages over standard optimization methods: it accommodates discrete and continuous variables, explores a wide design space, and handles multiple variables without necessitating objective function derivatives^27,28^.

**Figure 7:**
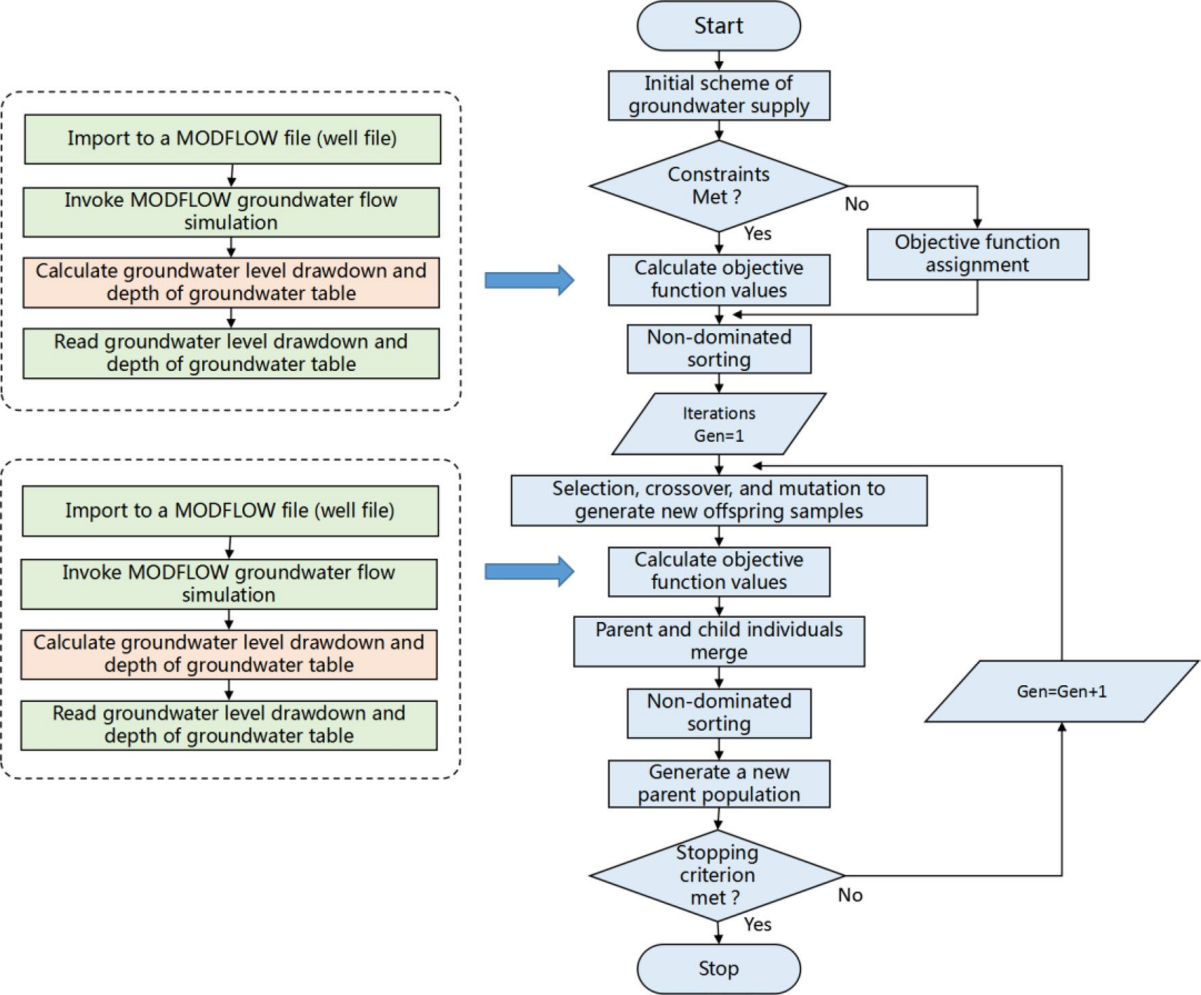
Flow chart of linked simulation model and GA-based optimization model.

In this study, a specialized software was created using the MATLAB environment. This software seamlessly integrates the flow-simulation model (MODFLOW) with the optimization technique (GA). The software’s structure is visually represented in Fig. 7. During the 2nd step, after the creation of the initial population, the resulting pumping rates are then matched to the actual wells. Subsequently, this specialized software generates input files for MODFLOW and executes the simulation model to calculate hydraulic heads at the well positions. The differences between initial and simulated hydraulic head values are recorded as water-level drops. Equation (4) defines the objective function, total cost, for the optimization model. Additionally, the software formulates and enforces any necessary constraints before initiating the optimization algorithm. As required, outcomes from the optimization model interact with the simulation model. This iterative process continues until the maximum number of iterations of the GA is reached. In the optimization process, the population size was defined as 100, the initial population was 100 groups of randomly generated decision variables, the crossover probability was 0.5, the mutation probability was 0.5, and the largest genetic iteration number was defined as 500.

## Results and discussion

Initially, the simulation–optimization model was employed to predict the groundwater level fluctuations in accordance with the existing emergency water supply schemes. Subsequently, proposed schemes were optimized with the aim of minimizing withdrawal costs while fulfilling current water demands. These schemes include scheme I (optimization of pumping rates), scheme II (joint of optimization of pumping rates and number of wells) and scheme III (joint of optimization of pumping rates, number of wells and well locations). Ultimately, the optimal emergency water supply scheme was determined, taking into account factors such as the area of the groundwater level depression funnel, the thickness of dewatering aquifer and the recovery of the groundwater level.

### Existing emergency water supply scheme

**Figure 8:**
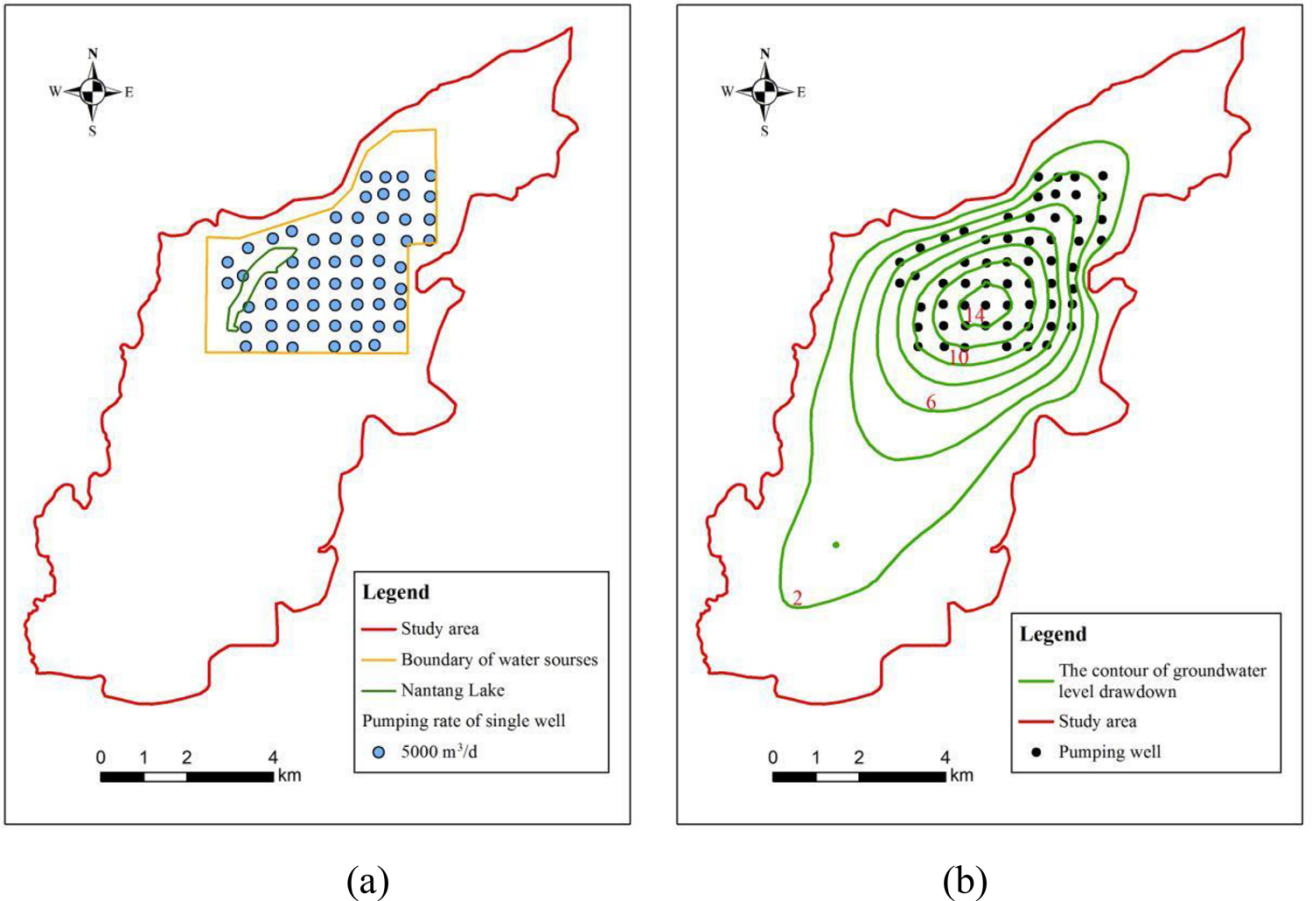
(**a**) Spatial distribution of groundwater exploitation wells. (**b**) Contour map of groundwater level drawdown after 3 months of water supply period.

In the existing scheme, the youkou emergency water source field is equipped with 60 uniformly spaced pumping wells, each with a distance of approximately 400 m and a single well pumping rate of 5000 m^3^/day. The planned emergency water supply duration spans 3 months, aiming to provide 300,000 m^3^/day (Fig. 8a). The prediction results are illustrated in Fig. 8b, which portrays the contour of groundwater level drawdown following a 3-month water supply period. Notably, the maximum groundwater level drawdown within the youkou emergency water source field reached 18.17 m, yet the aquifer remained undrained. After the 3-month water supply period, the dewatered aquifer thickness at the central depression was 15.19 m, constituting more than half (55.81%) of the initial aquifer thickness.

Overall, the existing scheme successfully met the demand for emergency water supply. Despite this accomplishment, there was still substantial groundwater level drawdown, and efforts need to be made to address this concern.

**Figure 9:**
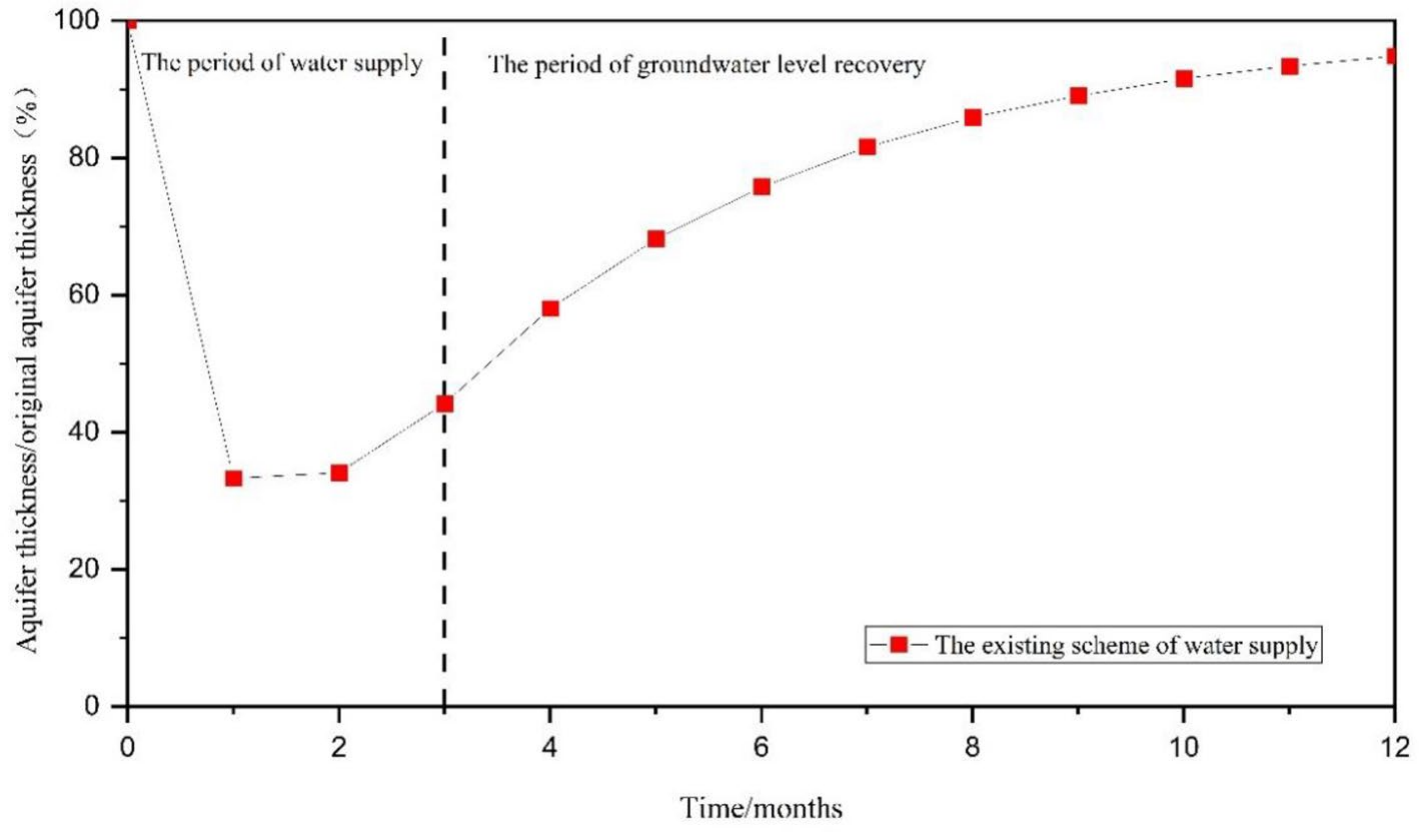
The ratio of the central aquifer’s thickness within the funnel to the initial aquifer’s thickness.

Following the cessation of the emergency water supply, there has been a gradual recovery of groundwater level, leading to a restoration of the aquifer’s thickness to 94.84% of the pre-supply levels after nine months of ceasing pumping. The aquifer has a strong ability to recover, and the groundwater level has essentially returned to its initial state (Fig. 9). The analysis results indicate that the youkou water source field presents some potential for emergency water supply. However, it should be noted that the drained aquifer thickness currently stands at more than half of its initial levels, emphasizing the necessity for optimizing existing emergency water supply scheme.

### Optimization results of water supply schemes

#### Objective function analysis

**Figure 10:**
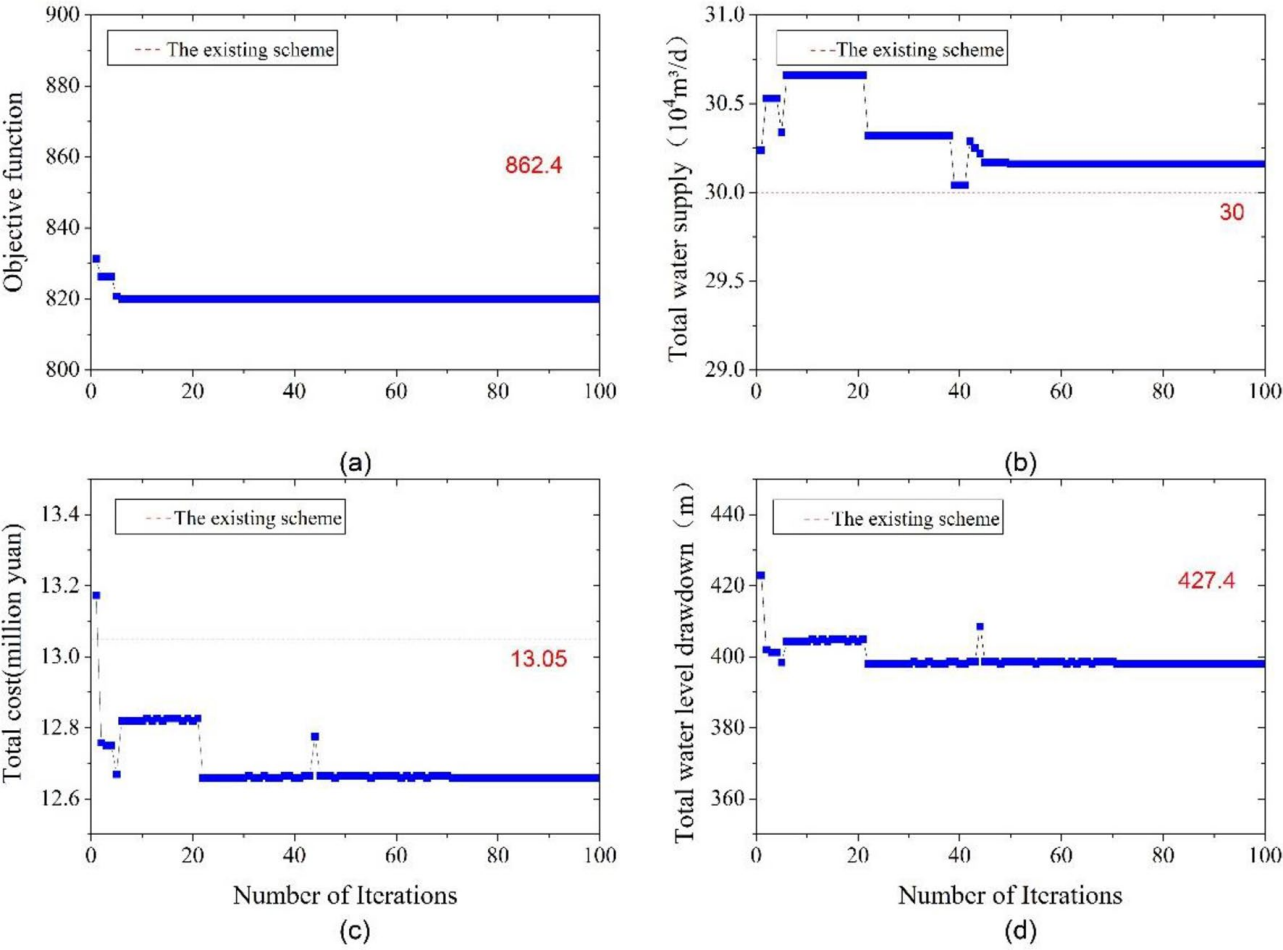
Convergence diagram for optimization scheme I: (**a**) objective function; (**b**) total water supply; (**c**) total cost; (**d**) groundwater drawdown of wells.

**Figure 11:**
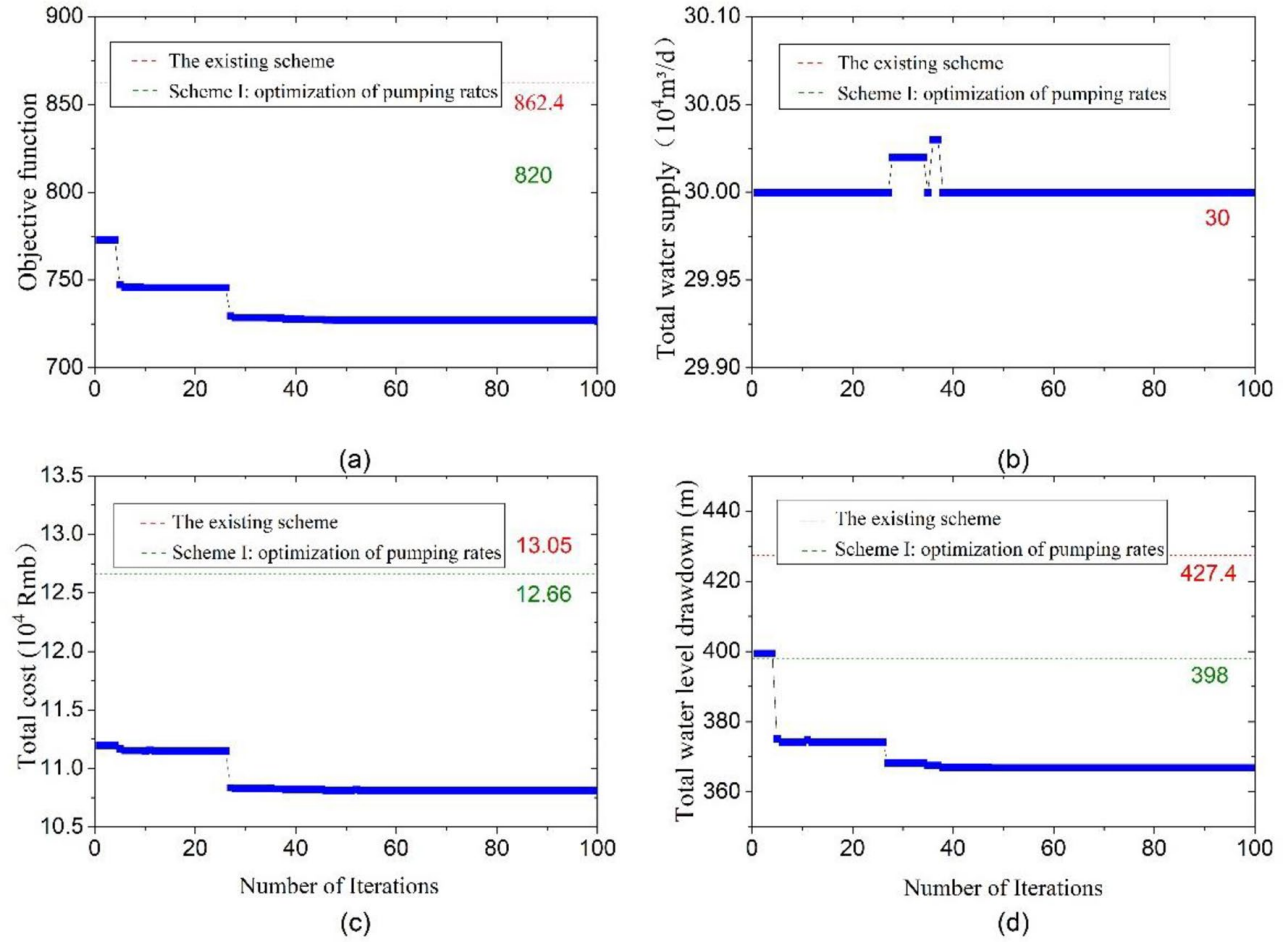
Convergence diagram for optimization scheme II: (**a**) objective function; (**b**) total water supply; (**c**) total cost; (**d**) groundwater drawdown of wells.

**Figure 12:**
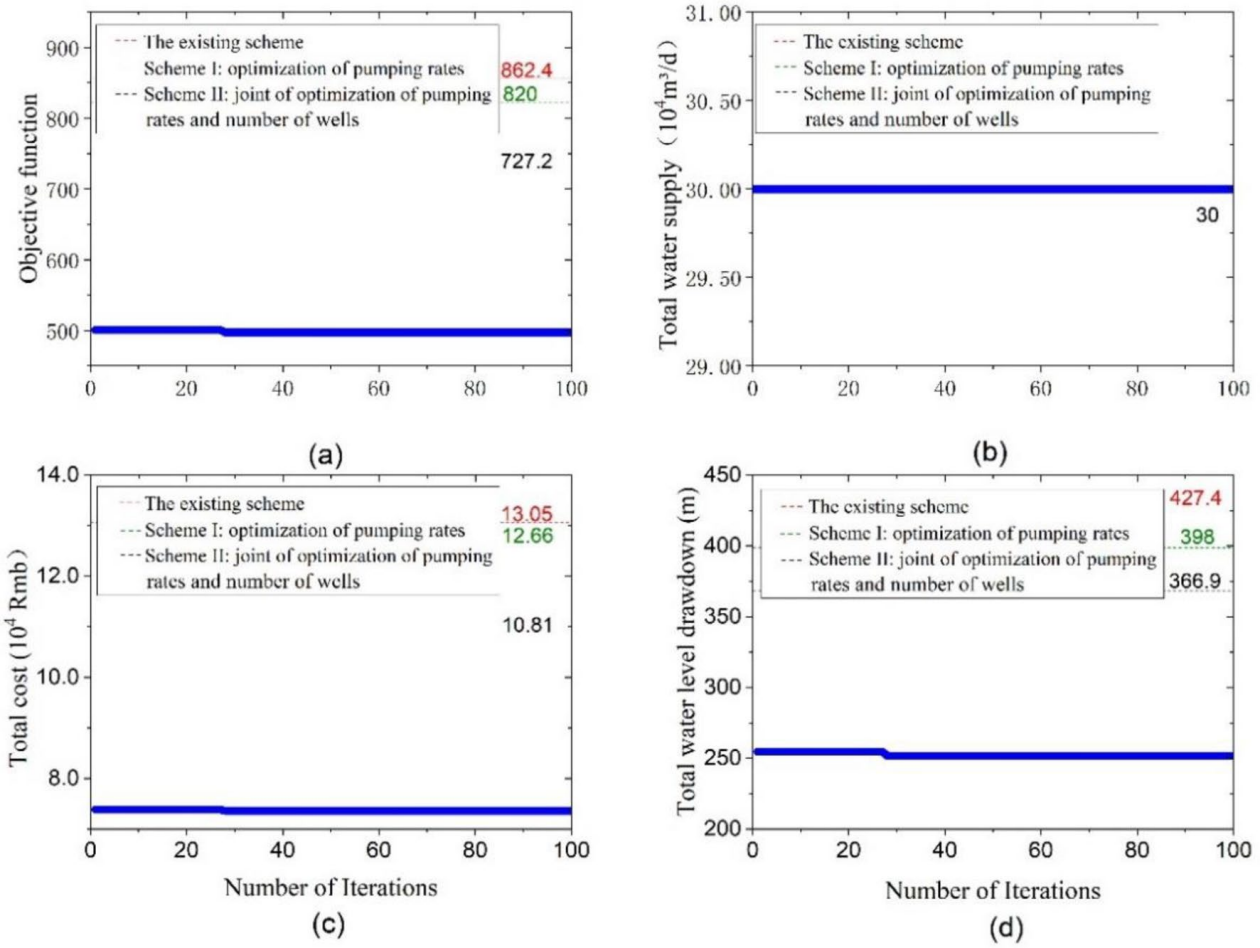
Convergence diagram for optimization scheme III: (**a**) objective function; (**b**) total water supply; (**c**) total cost; (**d**) groundwater drawdown of wells.

For the proposed optimization schemes, the trend of convergence for the best value of the objective function, total water supply, groundwater drawdown of wells, and total cost was illustrated in Figs. 10, 11 and 12, respectively.

Figure 10 illustrates that after 22 iterations, the model achieves convergence to the optimal solution with an objective function value for optimization scheme I approximately 4.92% lower than that of the existing scheme. Furthermore, the total cost of construction and operation declined from 13.05 × 10^4^ to 12.66 × 10^4^ CNY, indicating a reduction of around 3%. Additionally, the overall groundwater drawdown of the monitoring wells decreased from 427.4 m in the existing scheme to 398.02 m.

Figure 11 illustrates the model’s convergence to the optimal solution after 48 iterations, achieving an objective function value for optimization scheme II approximately 15.67% lower than that of the existing scheme. Moreover, the total cost of construction and operation experienced a significant decrease, from 13.05 × 10^4^ to 10.81 × 10^4^ CNY, marking a reduction of 17.16%. Additionally, the overall groundwater drawdown of the monitoring wells declined from 427.4 m in the existing scheme to 366.85 m.

Figure 12 illustrates that the model converged to the optimal solution after 28 iterations, with the value of the objective function for the optimized scheme III being about 42.35% lower than that of the existing scheme. Moreover, the total cost of construction and operation experienced a significant decrease, from 13.05 × 10^4^ to 7.36 × 10^4^ CNY, marking a notable reduction of 43.60%. Additionally, the overall groundwater drawdown of the monitoring wells decreased dramatically from 427.4 m in the initial scheme to 251.81 m.

#### Pumping rates analysis

**Figure 13:**
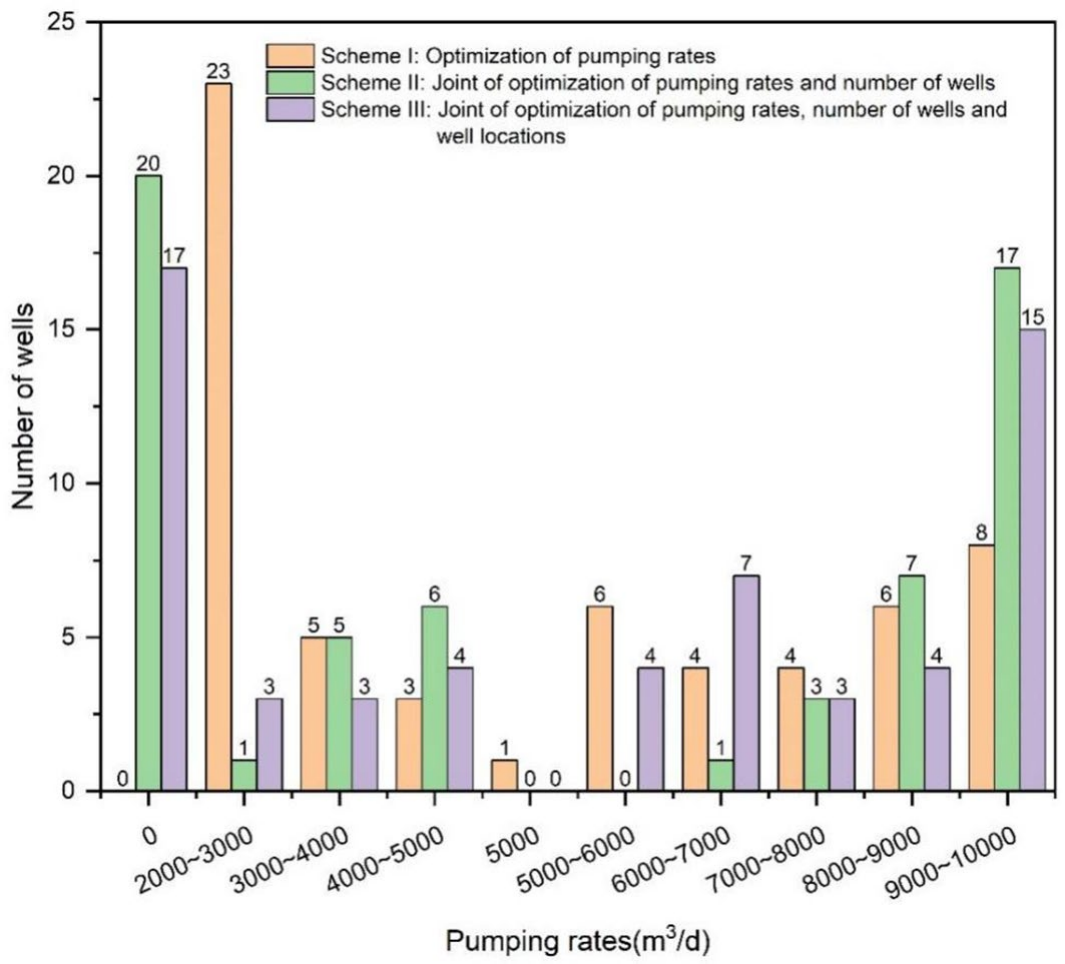
Statistics of single well pumping rate under the optimization schemes.

Figure 13 illustrates the pumping rates of a single well for the three optimization schemes. For the scheme of optimization of pumping rates, the pumping rate increased for 28 wells, while decreased for 31 wells, andremained at 5000 m^3^/day for one well. When pumping rates and number of wells were jointly optimized, the number of wells after optimization was 40. In comparison to the existing scheme, the pumping rate increased for 28 wells, while decreased for 12 wells. When the pumping rates, number of wells, and well locations were optimized together, the number of wells after optimization was 43, with the pumping rate of 10 wells below 5000 m^3^/day and that of 33 wells above 5000 m^3^/day.

**Figure 14:**
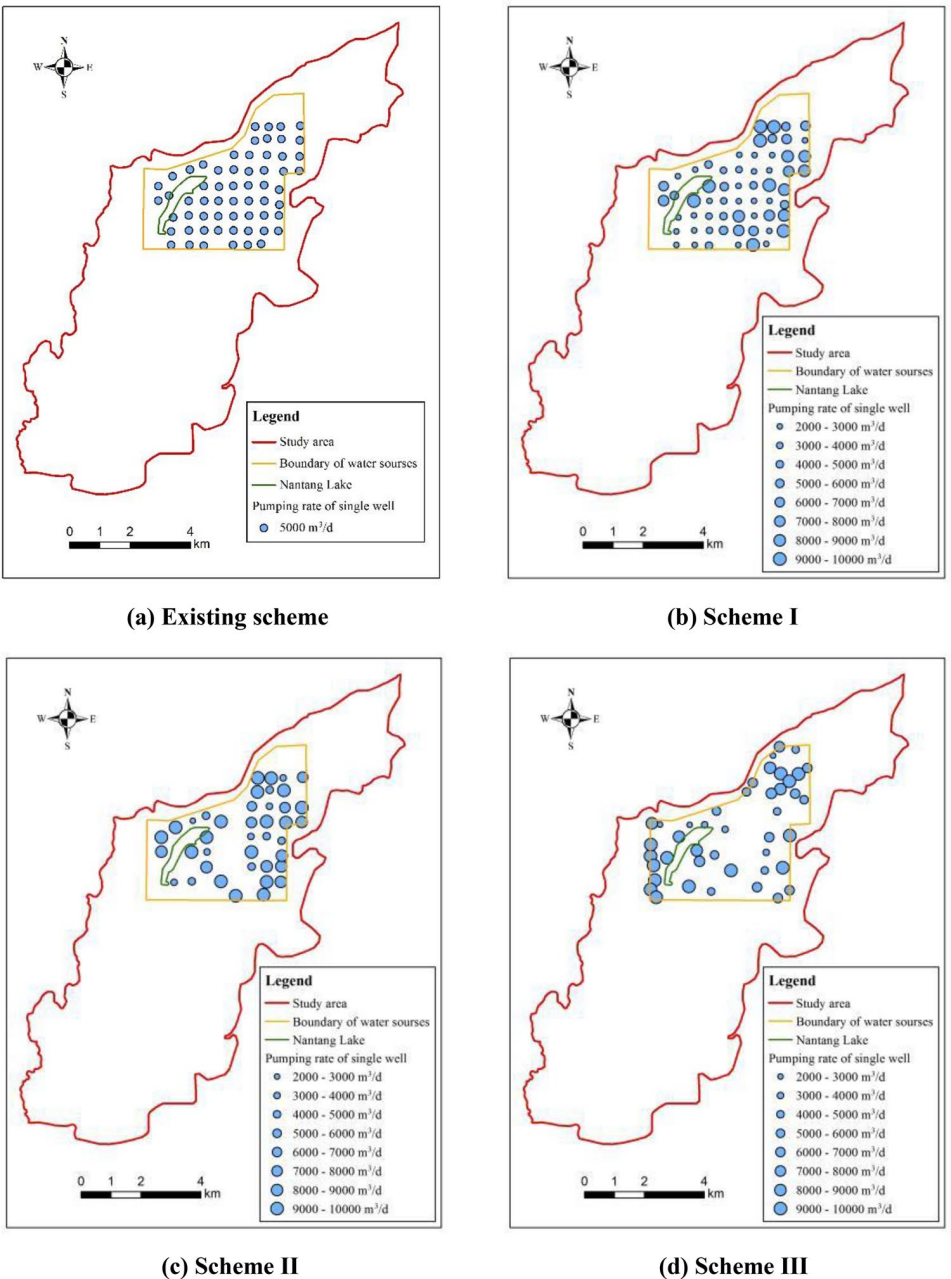
Spatial distribution of groundwater exploitation wells for the optimization schemes.

Figure 14 displays the locations of these wells in comparison to the existing wells. For scheme I, the wells with high pumping rates are located in the northern, eastern, southeastern and northwestern regions of the water source field. The distribution is designed to minimize the drop in the water level of the monitoring wells, leading to lower environmental costs for achieving the water supply target. The overall level of water source operation is lower and the operation cost investment is reduced, so the optimized solution is better than the existing solution (Fig. 14b).

For scheme II, active wells are primarily concentrated in the northern, eastern, and western regions of the pumping area, with sporadic distribution in the southern part. This distribution corresponds to the pattern of wells with higher pumping rates in the optimized scheme I. In the northern part of the pumping area, where the initial water level is higher, and the thickness of the Quaternary aquifer is substantial, the resulting groundwater level drawdown from pumping is relatively small. In the eastern region, the hydraulic conductivity is relatively low, and it is situated very close to the eastern lateral recharge boundary, facilitating lateral groundwater inflow recharge. Conversely, the western part of the pumping area has fewer pumping wells, mainly scattered around the water body perimeter and southwestern region, resulting in a relatively minor impact on the water level drawdown in monitoring wells (Fig. 14c).

The optimization results of scheme III reveal a concentration of wells with higher pumping rates in the northern regions of the water source field. This concentration is due to the relatively large aquifer thickness in that area, which is distant from the monitoring holes (Fig. 14d). Consequently, the reduction in water level in the monitoring holes is relatively minimal when compensating for groundwater exploitation. Numerous pumping wells are distributed along the northwest boundary, yet the pumping rate of each individual well remains relatively low. This is attributed to the proximity of this area to the river boundary, allowing for favorable access to water recharge from the south branch of Ganjiang. However, the presence of powdered and silty clay layers in the Ganjiang riverbed restricts the recharge of Ganjiang to the aquifer. With the introduction of the decision variable for the pumping well locations, the distribution of pumping wells becomes more concentrated in areas characterized by high aquifer thickness and improved recharge conditions.

#### Groundwater drawdown analysis

**Figure 15:**
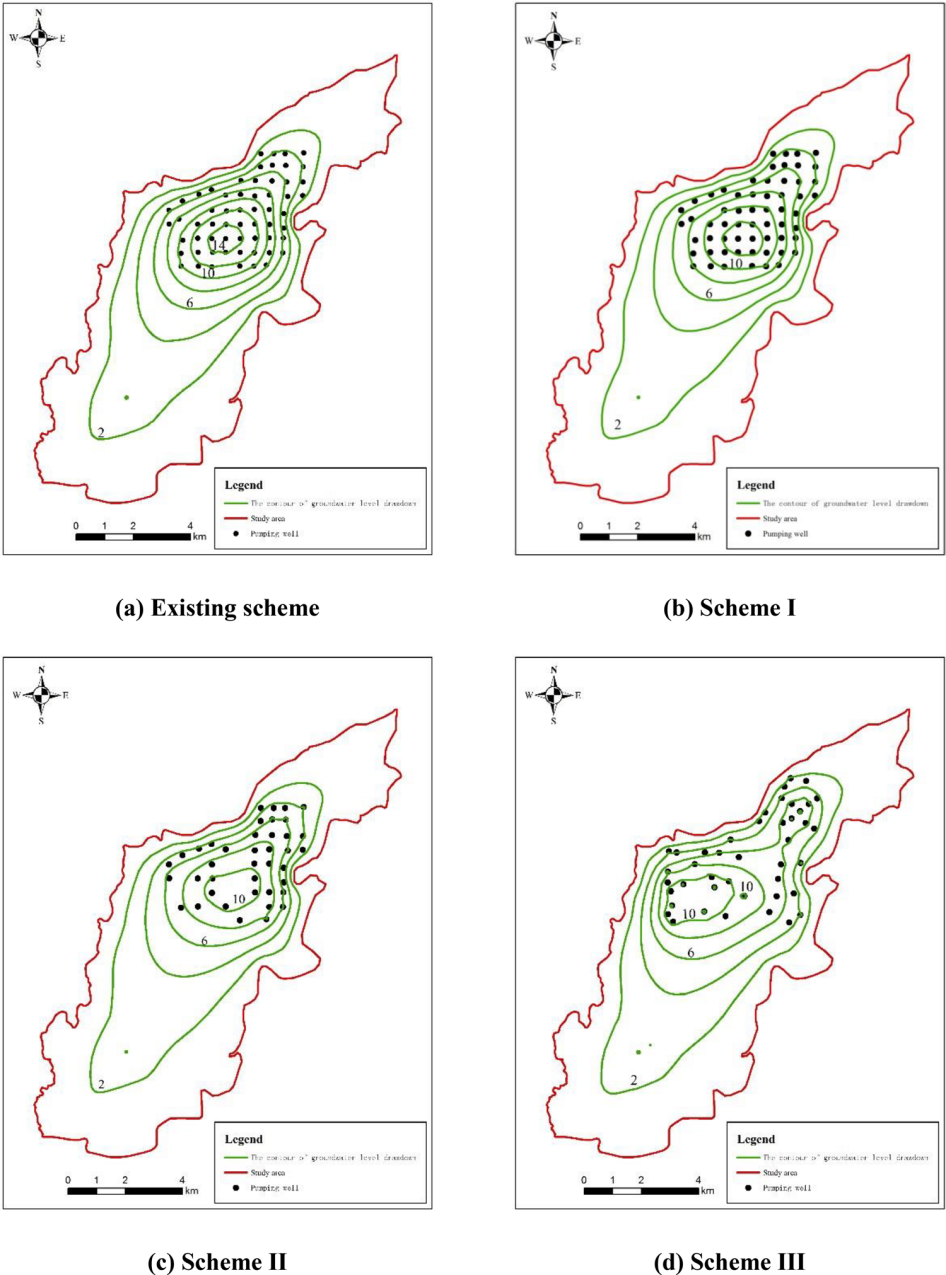
Contour maps of groundwater level drawdown after three months of water supply.

For the optimization scheme I, scheme II and scheme III, the contours of groundwater level drawdown after 3 months of water supply were shown in Fig. 15. Taking the 6 m drawdown contour as the boundary line of the funnel, the area of groundwater depression funnel in scheme I was reduced from 15.98 to 14.50 km^2^ comparing with the existing scheme, and the maximum drawdown of groundwater level was 15.78 m. Besides, the area of groundwater depression funnel in scheme II was reduced from 14.50 to 13.30 km^2^ comparing with the optimization scheme I, and the maximum drawdown of groundwater level was 12.05 m. Moreover, the area of groundwater depression funnel in scheme III was raised from 13.30 to 15.20 km^2^ comparing with the scheme II, and the maximum drawdown of groundwater level was 11.72 m.

#### Groundwater level recovery analysis

**Figure 16:**
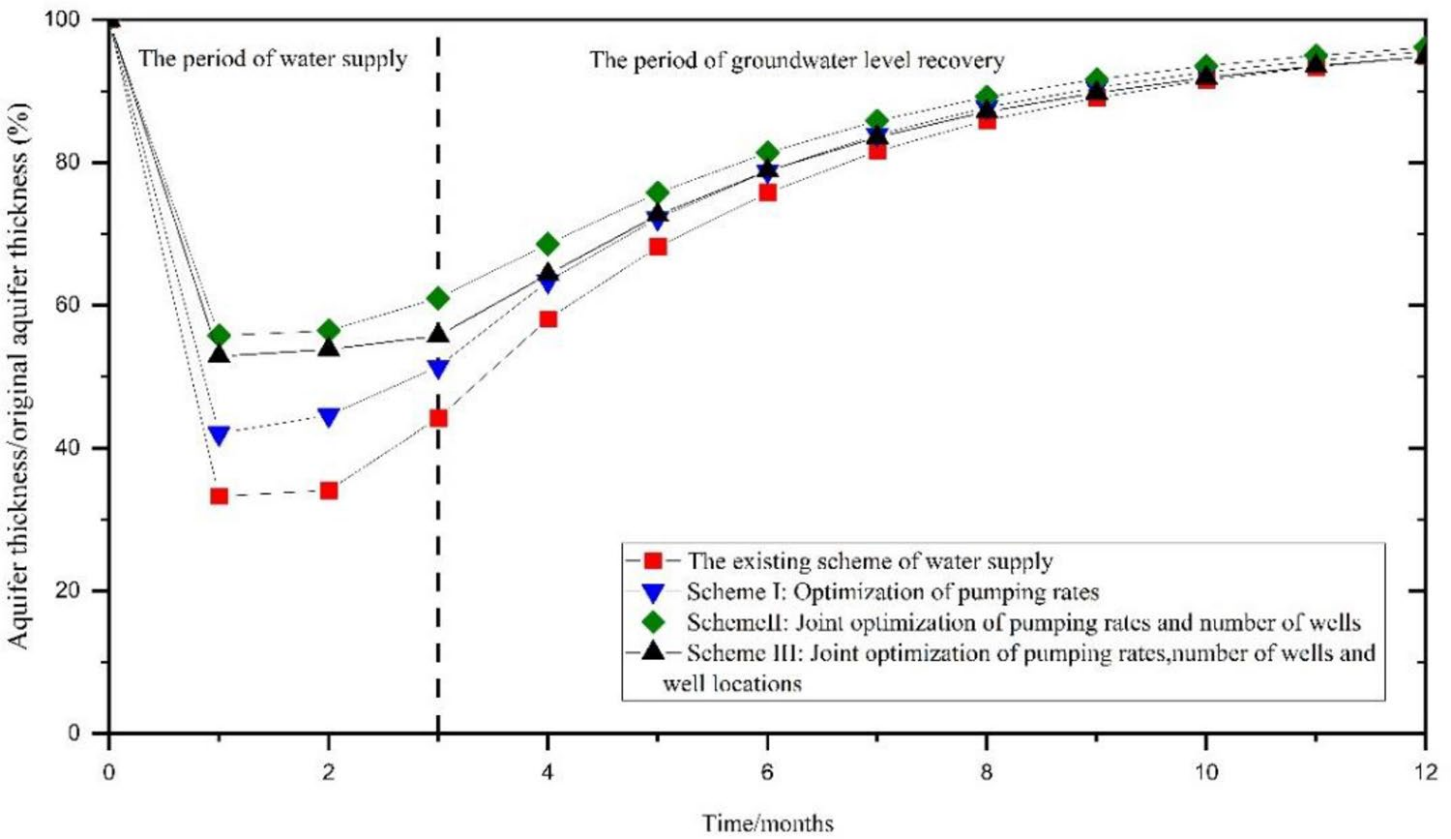
The ratio of the central aquifer’s thickness within the funnel to the original aquifer’s thickness.

In schemes I through III, the aquifer’s dewatered thickness at the center of the funnel was 13.24 m, 10.62 m, and 11.01 m, respectively. This corresponded to a reduction of 48.62%, 39.01%, and 44.29% in comparison to the initial thickness before the emergency water supply (Fig. 16).

Following the cessation of the emergency water supply, the groundwater level exhibited a gradual recovery. In scheme I, the thickness of the aquifer reached 95.55% of its initial thickness, and the groundwater level essentially returned to its initial state after nine months. Moreover, the environmental impacts of the optimized scheme were less extensive than those of the original scheme. For scheme II, the thickness of the aquifer accounts for 96.11% of the initial thickness , and the groundwater level essentially returned to its initial state. Introducing a decision variable for the number of pumping wells amplified the optimization’s effect and reduced the potential for geological environmental issues. The scheme III exhibited a thickness of 94.83% of the initial thickness, and a groundwater level that essentially returned to its initial state. In contrast to scheme II, the incorporation of a decision variable for the pumping well locations slightly diminished the optimization’s impact and enhanced the mitigation of geological environmental concerns.

## Conclusions

In this paper, a transient simulation model characterizing groundwater flow was constructed and calibrated. The flow model was then used in conjunction with a genetic algorithm based optimization model to explore the optimal pumping schemes that meet current water demands while minimizing the cost of withdrawal. The specific conclusions and results are as follows:

1. The numerical three-dimensional transient groundwater flow model could correctly portray the structural characteristics of the aquifer with good reliability. The simulated water level is basically consistent with the dynamic change trend of the monitored water level, and the nodes with water level fitting error less than 0.5 m accounted for more than 84% of the number of nodes with known water level.The accuracy of the groundwater flow model can meet the simulation and prediction requirements.
2. Following an identification and verification process, the groundwater flow model was employed to simulate and predict the existing water supply scheme. The results show that after three months of emergency water supply, a depression funnel was formed with a maximum drop depth was 18.17 m. And the aquifer thickness recovered to 94.84% of its initial thickness after 9 months of stopping water supply.
3. With 300,000 m^3^/day as the emergency water supply target, the proposed schemes include scheme I (optimization of pumping rates), scheme II (joint of optimization of pumping rates and number of wells) and scheme III (joint of optimization of pumping rates, number of wells and well locations) were optimized and converged to the optimal solution at iterations 22, 48, and 28, respectively, with the objective function values reduced by about 4.92%, 15.67%, and 42.35%, indicating that the simulation optimization method has a good optimization effect.
4. The optimal water supply scheme is determined to jointly optimize the number of pumping wells and the pumping rates of a single well by comprehensively considering such factors as the area of the water level depression funnel, the dewatering thickness of the aquifer and the recovery of the groundwater level.

## Data Availability

The datasets used and/or analyzed during the current study available from the corresponding author on reasonable request.
